# Causal associations of iron status and back pain risk: A Mendelian randomization study

**DOI:** 10.3389/fnut.2022.923590

**Published:** 2022-08-10

**Authors:** Yidan Tang, Jiahui Wu, Mingzhe Xu, Tao Zhu, Yalan Sun, Hai Chen, Lining Wu, Chan Chen

**Affiliations:** ^1^Department of Anesthesiology and National Clinical Research Center for Geriatrics, Laboratory of Anesthesia and Critical Care Medicine, Translational Neuroscience Center, West China Hospital, Sichuan University, Chengdu, China; ^2^The Research Units of West China, Chinese Academy of Medical Sciences, Chengdu, China; ^3^Department of Respiratory and Critical Care Medicine, Targeted Tracer Research and Development Laboratory, West China Hospital, Sichuan University, Chengdu, China

**Keywords:** iron status, back pain, Mendelian, SNP, cause-effect

## Abstract

**Background:**

Observational studies have previously suggested a link between iron status makers and back pain. We conducted a two-sample Mendelian randomization (MR) study to determine the putative causal relationship between systemic iron status and back pain.

**Materials and methods:**

In this MR study, a genome-wide association study (GWAS) involving 48,972 individuals was used to identify genetic instruments highly associated with systemic iron status. The outcome data (back pain) were derived from the Neale Lab consortium’s summary data from the UK Biobank (85,221 cases and 336,650 controls). With the inverse variance weighted (IVW) method as the main analysis, conservative analyses (selecting SNPs with concordant change of iron status biomarkers) and liberal analyses (selecting SNPs with genome-wide significant association with each iron status biomarker) were carried out. For sensitivity analyses, the MR-Egger, MR-Egger intercept, weighted median, weighted mode, and MR based on a Bayesian model averaging approaches were used. The Cochran’s Q-test was used to detect heterogeneity.

**Results:**

Back pain was associated with genetically instrumented serum iron (OR = 1.01; 95% CI = 1.00–1.02, *p* = 0.01), ferritin (OR = 1.02; 95% CI = 1.00–1.04, *p* = 0.02), and transferrin saturation (OR = 1.01; 95% CI = 1.00–1.01, *p* = 0.01). Furthermore, there was no evidence of a link between transferrin and the risk of back pain (OR = 0.99, 95% CI = 0.98–1.00, *p* = 0.08). The sensitivity analyses and Cochran’s Q-test indicated that no pleiotropy or heterogeneity was detected (all *p* > 0.05).

**Conclusion:**

We provided potential genetic evidences for the causal associations of iron status with increased incidence of back pain. However, the evidences were weakened due to the low power. Further larger MR studies or RCTs are needed to investigate small effects.

## Introduction

Back pain (low back or neck pain with or without radicular symptoms) and osteoarthritis are the leading causes of disability across the world (1, 2). Clinical and epidemiological studies across 195 countries reported that low back pain could seriously affect the quality of life, which continues to be the leading cause of years lived with disability globally (568.4 million) (3, 4). One systematic review of 165 studies reported the point prevalence of back pain was 11.9% (Standard error of mean as 2), and the 1-month prevalence was 23.2% (Standard error of mean as 2.9) (5). Back pain could cost billions of dollars in economic costs (6, 7) and intangible expenses such as difficulty with household duties, caregiving, and participation in recreational activities, relationship problems, despair, and anxiety (8). Due to the high prevalence and heavy burden of back pain globally, it is critical to discover modifiable risk factors (9).

Iron is essential for cell survival, differentiation, protein synthesis, hormone production, and crucial components of cellular energy metabolism throughout brain development and growth (10, 11). However, low iron levels cause erythropoiesis to be limited, resulting in anemia. High amounts of labile iron, on the other hand, are very hazardous to cells because they produce reactive oxygen species that can harm cells and organs (12). The iron homeostasis dysregulation is often associated with multiple pathologies, including aging in humans (13), and neurodegenerative diseases such as Alzheimer’s disease (14), and Parkinson’s disease (15). A case-control study reported all serum levels of minerals including Fe were significantly different in low back pain patients compared to healthy individuals (16). A strong link between serum iron and the severity of low back pain was reported (16). Intervertebral disc degeneration has been linked to iron overload as a predominantly triggered condition for low back pain (17).

In the last decade, low back pain genetic determinants have gotten more attention (9). Gene polymorphism is widespread in biological populations, resulting in differences and diversity among biological populations. In recent years, it has been found that gene polymorphism is the basis of affecting pain sensitivity and the fundamental reason for the difference in individual efficacy and adverse reactions at the standard dose of analgesics (18, 19). However, the causal associations between systemic iron status and back pain remain unclear. A significant drawback of observational research is the difficulty in distinguishing between true causal connections and spurious associations owing to confounding and reverse causation (20). In this instance, Mendelian randomization (MR) has been applied to examine the specific causal relationship (21, 22). Because genotypes occur before illness onset and are usually unaffected by postnatal lifestyle or environmental influences, the MR technique can minimize ambiguity and eliminate reverse causation bias (23).

In this study, we performed a two-sample MR study to explore the potential causality between iron status and incidence of back pain.

## Materials and methods

### Study design

**FIGURE 1:**
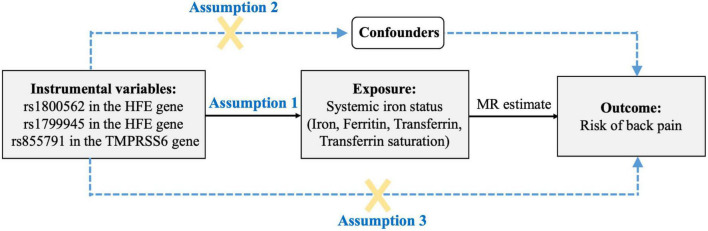
The design flow chart for the Mendelian randomization (MR) study. Assumption 1: Genetic instrumental variables are associated with systemic iron status with the genome-wide significance; Assumption 2: Genetic instrumental variables are not associated with any measured and unmeasured confounders; Assumption 3: Genetic instrumental variables do not influence the risk of back pain through other pathways. MR, Mendelian randomization.

For the present study, we used a two-sample MR to estimate the associations of systemic iron status with back pain risk. A total of four iron status biomarkers were considered as exposures. The selected SNPs are strongly associated with exposures at the genome-wide significance (*p* < 5 × 10^–8^). In addition, all SNPs were restricted in linkage equilibrium (pairwise *r*^2^ ≤ 0.01). The overview of our study design is shown in Figure 1.

### Systemic iron status instruments

The largest genome-wide association study (GWAS) analysis was conducted previously by the Genetics of Iron Status (GIS) consortium to derive association estimates between Single Nucleotide Polymorphisms (SNP) and iron status (24). The meta-analysis merged data from 11 discovery cohorts and eight replication cohorts, with 48,972 European participants. Specifically, serum iron, ferritin (log-transformed), transferrin saturation, and transferrin were employed for iron status biomarkers. The details of included GWAS are shown in Table 1.

**TABLE 1:** Details of GWAS studies.

Phenotypes	Consortium	Population	Sample size
Back pain	Neale lab	European	336,650
Iron status	Genetics of Iron Status (GIS)	European	48,972

### Genetic associations with back pain

Genetic associations with back pain were derived from UK Biobank summary data made by the Neale Lab consortium, which includes 336,650 unrelated individuals of European ancestry and adjusted for age, age^2^, sex, the first 20 principal components, the interactions of age^2^ with age, and sex.^1^ Details were collected through a specific pain-related questionnaire, which included the question, “In the last month, have you experienced any of the following that interfered with your usual activities?” The options were: (1) headache; (2) facial pain; (3) neck or shoulder pain; (4) back pain; (5) stomach or abdominal pain; (6) hip pain; (7) knee pain; (8) pain all over the body; (9) none of the above; (10) prefer not to say. The aggregated GWAS results were involved 85,221 cases and 336,650 controls. The details were shown in Supplementary Table 1.

### Mendelian randomization analysis

The two-sample MR method was employed for the primary MR analysis. Causal associations between systemic iron status and risk of back pain were estimated based on the inverse variance weighting (IVW) model. The random-effect model was adopted in the IVW analysis. A threshold of *p* less than 0.05 was utilized to determine statistical significance. We used the formula, F = R^2^ × (N–k–1)/[(1–R^2^) × k], to generate the F statistic, where R^2^ is the percentage of iron status variability explained by each SNP, N is the sample size of GWAS for iron status, and k is the number of SNPs (25). The IVW model’s *a priori* power estimate was also performed. Specifically, we used an online calculator to evaluate the observable effects of iron status on the probability of back discomfort at least 80% statistical power threshold (26).

### Sensitivity analysis

Sensitivity analyses were performed using the MR Egger (27), Weighted median (WM) (28), and Weighted mode techniques to account for the likelihood of pleiotropy bias. Among these tactics are pleiotropic or defective instruments, which are more resistant to possible breaches of the standard instrumental variable assumption. The WM estimate, as the WM of the SNP-specific estimations, produces correct findings if SNPs contributing 50% of the weight are trustworthy instruments (28). MR Egger regression also estimates the underlying causative impact, even if all genetic variations are invalid. Nevertheless, MR Egger can be inaccurate, especially when the estimates are similar, or the number of genetic instruments is small. These SNPs’ potential pleiotropic effects were assessed using MR-Egger regression, in which the slope reflects causal estimates adjusted for pleiotropy and the intercept represents the average pleiotropic effects of all SNPs. SNP heterogeneity was assessed using the Cochran Q statistic. The connection between iron status and back pain was represented by odds ratios (ORs) with 95% confidence intervals (CIs) per one standard deviation log-transformed genetically predicted increase in iron status. The leave-one-out analysis was conducted to determine whether a single SNP was responsible for the significant results.

### Mendelian randomization based on a Bayesian model averaging estimates

We performed a novel approach extending multivariable MR, Mendelian randomization based on a Bayesian model averaging (MR-BMA), based on the Bayesian model averaging method, to circumvent the limits of logistic regression approaches and prioritize the most causally related risk factors for back pain (29). MR-BMA considers all possible combinations of the four biomarkers of systemic iron status and generates posterior probability (PP) for each specific model (one risk factor or a combination of multiple risk factors) (29). Then, MR-BMA computes a marginal inclusion probability (MIP) for each iron risk factor, which means the sum of the PP over all models where the iron status is presented. Furthermore, MR-BMA will report the model-averaged causal effects (MACE) for each iron status biomarker by ranking all the iron status biomarkers according to the corresponding MIP. Finally, MR-BMA will prioritize the best models via the PP value. Invalid instruments were identified as outliers in terms of linear model fit using the *Q*-statistic, used to determine heterogeneity in the meta-analysis (30). The Cook’s distance (Cd) was used to quantify influential observations (31). We repeated the analysis while eliminating outliers (*Q* > 10) and Cd exceeding the threshold that was consistently discovered in all the best models (PP > 0.02).

The R program (version 4.1.2) and Two-Sample MR package (version 0.5.6) was used to conduct all the studies.

## Results

### Genetic variables for systemic iron status

Individual SNPs of iron status (39 to 3,340) had F statistics of more than 10, indicating that bias from weak instrumentals was unlikely (32). The changes of three SNPs, including rs1800562 (25), rs1799945[HFE gene], and rs855791[TMPRSS6 gene], were concordant with four iron status biomarkers (serum iron, ferritin, transferrin, and transferrin saturation). According to independent and LD analyses, the three SNPs are therefore employed for conservative analysis (Table 2). Furthermore, larger SNP groups due to the genome-wide significant association with iron status biomarkers were selected for liberal analysis (Table 2).

**TABLE 2:** Details of GWAS studies.

SNP	CHR/BP (Build 37)	Nearest Genes(s)	EA/OA	EAF	Beta (SE)			
					Iron	Ferritin (log)	Transferrin	Transferrin saturation
rs1800562	6/26,093,141	HFE	A/G	0.067	0.328 (0.016)	0.204 (0.016)	−0.479 (0.016)	0.577 (0.016)
rs1799945	6/26,091,179	HFE	C/G	0.85	−0.189 (0.01)	−0.065 (0.01)	0.114 (0.01)	−0.231 (0.01)
rs855791	22/37,462,936	TMPRSS6	A/G	0.446	−0.181 (0.007)	−0.055 (0.007)	0.044 (0.007)	−0.19 (0.008)
rs744653	2/190,378,750	WDR75—SLC40A1	T/C	0.854	0.004 (0.01)	−0.089 (0.01)	0.068 (0.01)	−0.028 (0.011)
rs8177240	3/133,477,701	TF	T/G	0.669	−0.066 (0.007)	0.021 (0.007)	−0.380 (0.007)	−0.380 (0.007)
rs9990333	3/195,827,205	TFRC	T/C	0.46	0.017 (0.007)	0.001 (0.007)	−0.051 (0.007)	0.039 (0.007)
rs7385804	7/100,235,970	TFR2	A/C	0.621	0.064 (0.007)	0.015 (0.007)	−0.003 (0.007)	0.054 (0.008)
rs4921915	8/18,272,466	NAT2	A/G	0.782	0.004 (0.009)	0.001 (0.009)	0.079 (0.009)	−0.026 (0.009)
rs651007	9/136,153,875	ABO	T/C	0.202	−0.004 (0.009)	−0.050 (0.009)	−0.001 (0.009)	−0.006 (0.009)
rs6486121	11/13,355,770	ARNTL	T/C	0.631	−0.009 (0.007)	0.006 (0.007)	−0.046 (0.007)	0.015 (0.008)
rs174577	11/61,604,814	FADS2	A/C	0.33	0.001 (0.007)	−0.012 (0.007)	0.062 (0.007)	−0.025 (0.008)
rs411988	17/56,709,034	TEX14	A/G	0.564	−0.002 (0.007)	−0.044 (0.007)	0.014 (0.007)	−0.012 (0.007)

### Associations of systemic iron status with back pain risk

**FIGURE 2:**
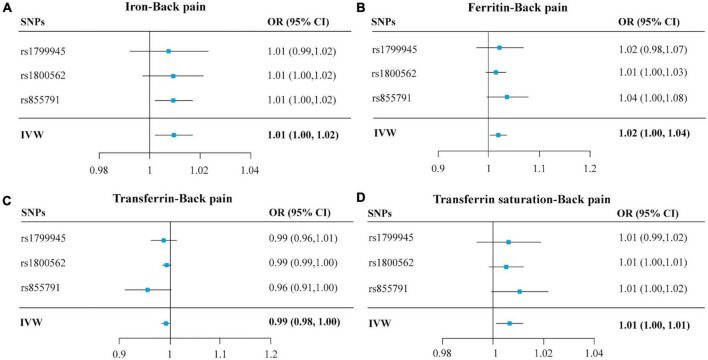
Forest plots summarizing the SNP-specific and overall MR estimates for the causal effects on back pain using the SNPs associated with four iron biomarkers in conservative analyses. Panels **(A–D)**, respectively, indicate the causal estimates of serum iron, ferritin, transferrin, and transferrin saturation on back pain. The horizontal lines represent the 95% CIs, while the solid black diamonds represent estimates of the causal effects of the genetic instruments. The gray diamond’s center indicates the overall MR estimate, while the diamond’s width indicates the 95% CIs. MR, Mendelian randomization; SNP, single nucleotide polymorphisms; OR, odds ratio; CI, confidence interval; IVW, inverse variance weighted.

**FIGURE 3:**
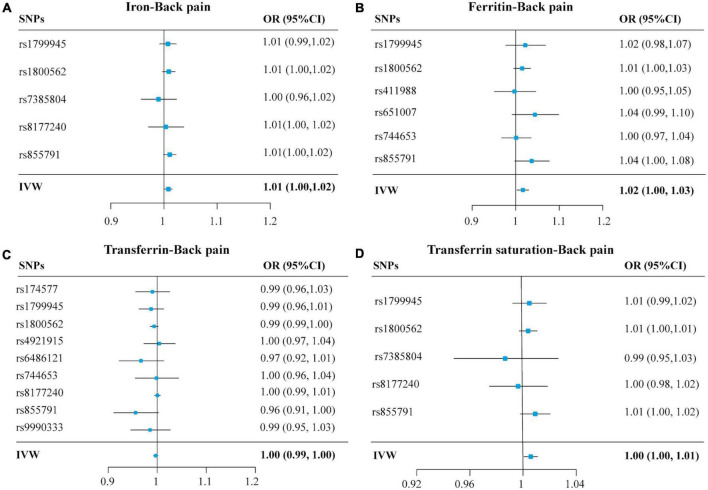
Forest plots summarizing the selective SNPs and overall MR estimates for the causal effects on back pain using the SNPs associated with four iron biomarkers in liberal analyses. Panels **(A–D)**, respectively, indicate the causal estimates of serum iron, ferritin, transferrin, and transferrin saturation on back pain. The horizontal lines represent the 95% CIs, while the solid black diamonds represent estimates of the causal effects of the genetic instruments. The gray diamond’s center indicates the overall MR estimate, while the diamond’s width indicates the 95% CIs. MR, Mendelian randomization; SNP, single nucleotide polymorphisms; OR, odds ratio; CI, confidence interval; IVW, inverse variance weighted.

The OR of back pain per SD unit increasing in each iron status biomarker revealed an association between increased iron status and the risk of back pain. For conservative analysis, our results determined by IVW method showed that the genetically predicted high levels of serum iron (OR = 1.01, 95% CI = 1.00–1.02; *p* = 0.01), log-transformed ferritin (OR = 1.02, 95% CI = 1.00–1.04; *p* = 0.02), and transferrin saturation (OR = 1.01, 95% CI = 1.00–1.01; *p* = 0.01) were significantly associated with back pain risk (Figures 2A,B,D and Supplementary Figure 1). But transferrin was no significant effect (OR = 0.99, 95% CI = 0.98–1.00; *p* = 0.08) (Figure 2C and Supplementary Figure 1). Investigating back pain risk using the separately selected SNPs by their genome-wide significant association with each iron status biomarker also produced directionally consistent results, as shown in Figure 3, Table 3, and Supplementary Figure 3. The minimum and maximum real causal effects (ORs) achieving at least 80% statistical power are presented in Supplementary Table 1.

**TABLE 3:** Associations between genetically instrumented systemic iron status and back pain using the separately selected SNPs associated with all four iron status biomarkers.

Exposure	Method	N SNP	Beta	SE	*P-*value	Egger intercept	Egger SE	Egger *P*-value	Cochran’s *Q*	*Q* DF	*Q P*-value
Serum iron	MR Egger	5	0.014	0.007	0.138	–0.001	0.001	0.475	0.295	1	0.587
	Weighted median	5	0.009	0.004	0.026						
	Weighted mode	5	0.009	0.004	0.098						
	Inverse variance weighted	5	0.008	0.004	0.018				1.466	2	0.481
Ferritin	MR Egger	6	0.011	0.013	0.465	0.001	0.001	0.525	0.128	1	0.721
	Weighted median	6	0.015	0.008	0.073						
	Weighted mode	6	0.014	0.01	0.218						
	Inverse variance weighted	6	0.016	0.007	0.016				0.982	2	0.612
Transferrin	MR Egger	9	0.0007	0.003	0.804	–0.0009	0.0006	0.123	4.892	7	0.673
	Weighted median	9	–0.0006	0.002	0.81						
	Weighted mode	9	–0.001	0.002	0.551						
	Inverse variance weighted	9	–0.003	0.002	0.208				7.961	8	0.437
Transferrin saturation	MR Egger	5	0.008	0.004	0.152	–0.0006	0.0009	0.566	1.614	3	0.656
	Weighted median	5	0.006	0.003	0.054						
	Weighted mode	5	0.006	0.003	0.118						
	Inverse variance weighted	5	0.006	0.003	0.025				2.029	4	0.731

### Sensitivity analysis provided no indication of unknown pleiotropy

Accordingly, the sensitivity analysis supported these effects for serum iron, ferritin, and transferrin saturation with a significant *p*-value obtained from the weighted median method (*p* < 0.05), as shown in Table 4 and Supplementary Figure 2. Besides, of four iron status biomarkers, all MR-Egger intercepts did not differ significantly from zero (*p* > 0.05), indicating that there was no directional pleiotropy for back pain (Table 4). Lastly, both and IVW and MR egger in Cochran’s *Q*-test showed *p* > 0.05 (Table 4), suggesting no significant heterogeneity of four iron status genetic IVs in back pain GWAS. Similarly, MR Egger intercepts utilizing the independently selected SNPs did not differ markedly from null (*p* = 0.525, 0.475, 0.123, and 0.566 for serum iron, ferritin, transferrin saturation, and transferrin, respectively), indicating that pleiotropy for back pain is not statistically significant (Table 3 and Supplementary Figure 4). Finally, the Cochran *Q* statistics (MR Egger and IVW method) using the separately selected SNPs showed low heterogeneities (*p* > 0.05) for serum iron, ferritin, and transferrin, transferrin saturation (Table 3). The leave-one-out sensitivity analysis revealed that eliminating one of the iron status SNPs had no effect on the results (Supplementary Figures 5, 6).

**TABLE 4:** Associations between genetically instrumented systemic iron status and back pain using the three SNPs associated with all four iron status biomarkers.

Exposure	Method	N SNP	Beta	SE	*P*-value	Egger intercept	Egger SE	Egger *P*-value	Cochran’s *Q*	*Q* DF	Q *P*-value
Serum iron	MR Egger	3	0.008	0.015	0.686	0.0003	0.003	0.936	0.122	1	0.727
	Weighted median	3	0.01	0.004	0.020						
	Weighted mode	3	0.01	0.005	0.201						
	Inverse variance weighted	3	0.009	0.004	0.011				0.132	2	0.936
Ferritin	MR Egger	3	0.008	0.015	0.688	0.001	0.001	0.525	0.128	1	0.721
	Weighted median	3	0.017	0.009	0.064						
	Weighted mode	3	0.016	0.009	0.24						
	Inverse variance weighted	3	0.019	0.008	0.017				0.982	2	0.612
Transferrin	MR Egger	3	–0.003	0.005	0.711	–0.002	0.001	0.362	0.174	1	0.676
	Weighted median	3	–0.007	0.004	0.071						
	Weighted mode	3	–0.007	0.004	0.26						
	Inverse variance weighted	3	–0.008	0.004	0.079				2.618	2	0.27
Transferrin saturation	MR Egger	3	0.003	0.006	0.697	0.001	0.002	0.61	0.146	1	0.702
	Weighted median	3	0.006	0.003	0.051						
	Weighted mode	3	0.005	0.003	0.242						
	Inverse variance weighted	3	0.007	0.003	0.014				0.64	2	0.726

### Mendelian randomization based on a Bayesian model averaging estimates

**FIGURE 4:**
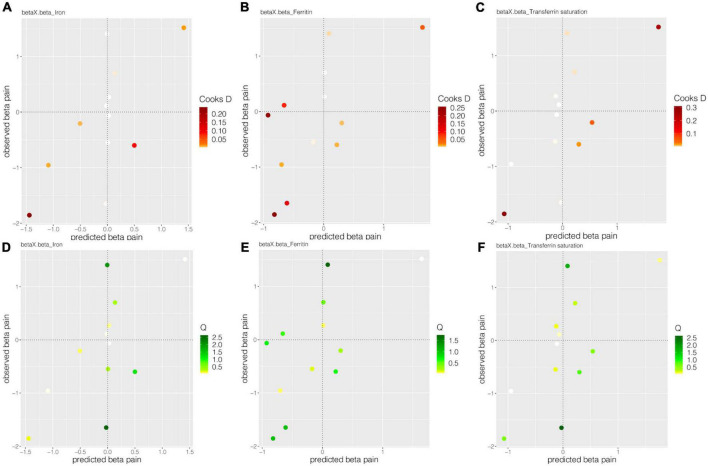
The Cooks D and *Q* estimate systemic iron status for back pain. Panels **(A–C)**, respectively, showed the Cook’s distances of 12 SNPs for the effect of iron, ferritin, and transferrin saturation on back pain. Panels **(D–F)**, respectively, show the *Q* statistic of 12 SNPs for the impact of iron, ferritin, and transferrin saturation on back pain.

For the MR-BMA analyses, 12 SNPs (rs1800562, rs1799945, rs855791, rs8177240, rs7385804, rs744653, rs651007, rs411988, rs9990333, rs4921915, rs6486121, and rs174577) were employed. Of selected 12 SNPs, all Cochran’s *Q* statistics were less than 10 (low heterogeneity), and Cd did not exceed the threshold (Figure 4, Table 5, and Supplementary Table 2). All the risk factors were then prioritized and ranked by their MIP, whereas, the best models were prioritized and ranked by their PP (Table 5). All the MR-BMA results were consistent with the MR results.

**TABLE 5:** Ranking of risk factors and models for back pain (MR-BMA analysis).

Risk factors/Model	Ranking by MIP	MIP	Averaged effect	Ranking by PP	PP	Causal estimate
Iron	2	0.293	0.002	2	0.291	0.009
Ferritin	1	0.518	0.008	1	0.515	0.016
Transferrin	3	0.182	0	4	0.01	–0.003
Transferrin saturation	4	0.011	0.001	3	0.18	0.006

MR-BMA, Mendelian randomization based on Bayesian model averaging; MIP, marginal inclusion probability; PP, posterior probability.

### Associations of back pain with systemic iron status

The set of MR analyses sought to assess the potential causative effect of back pain on systemic iron status. The findings revealed no significant causal association between back pain and systemic iron status (Table 6 and Supplementary Figure 7), which was consistent with the findings of sensitivity analyses (Table 6 and Supplementary Figure 8). Furthermore, no pleiotropy was detected in any of the analyses (Table 6).

**TABLE 6:** Mendelian randomization estimates for the causal effect of back pain on iron status biomarkers.

Outcome	Method	N SNP	Beta	SE	*P*-value	Egger intercept	Egger SE	Egger *P*-value	Cochran’s *Q*	*Q* DF	Q *P*-value
Serum iron	MR Egger	5	–3.759	5.042	0.510	0.035	0.041	0.460	1.483	3	0.686
	Weighted median	5	0.648	0.844	0.443						
	Weighted mode	5	0.624	1.126	0.609						
	Inverse variance weighted	5	0.464	0.654	0.479				2.196	4	0.700
Ferritin	MR Egger	5	5.229	4.703	0.347	–0.039	0.039	0.390	2.879	3	0.411
	Weighted median	5	0.908	0.798	0.255						
	Weighted mode	5	1.109	1.023	0.34						
	Inverse variance weighted	5	0.502	0.613	0.413				3.907	4	0.419
Transferrin	MR Egger	5	–3.607	5.156	0.535	0.031	0.042	0.520	1.265	3	0.738
	Weighted median	5	0.071	0.821	0.931						
	Weighted mode	5	0.109	0.672	0.871						
	Inverse variance weighted	5	0.412	1.103	0.728				1.793	4	0.774
Transferrin saturation	MR Egger	5	–0.145	5.067	0.979	0.003	0.041	0.940	1.492	3	0.684
	Weighted median	5	0.469	0.805	0.560						
	Weighted mode	5	0.721	1.101	0.549						
	Inverse variance weighted	5	0.268	0.657	0.683				1.498	4	0.827

## Discussion

Few studies have reported the relationship between systemic iron status and back pain. To assess the causal association between systemic iron status and back pain, we used a two-sample MR analysis. According to our MR assumptions, instrumental variables (SNPs) should only be associated to the outcome (back pain) through exposure (systemic iron status). Our results revealed that several genetically determined markers of systemic iron status are potentially associated with the risk of back pain.

Iron, a vital trace element, is required for a wide range of biological functions in all living species. However, excess iron causes oxidative stress and tissue damage in aerobic circumstances (33). Iron metabolism is be involved in the onset and pathological process of chronic diseases, including metabolic disorders and arthropathy (34, 35). Higher serum iron levels, transferrin saturation, ferritin, and lower amounts of transferrin, are associated with higher systemic iron status (36). The previous study has linked the disturbances of iron homeostasis to various pain-related consequences (37). Iron overload is typically detected by measuring serum transferrin saturation (the amount of ferric iron bound to the carrier protein transferrin in the circulation; >45 percent indicates iron excess) in conjunction with serum ferritin concentration (35). Iron overload has been shown to hasten bone loss in healthy postmenopausal women and middle-aged men, increase apoptosis, and impair chondrocyte functional competence (38, 39). Furthermore, aberrant iron accumulation causes nerve injury in rats by up-regulating CXCL10/CXCR3 signaling in the spinal dorsal horn, resulting in mechanical allodynia and thermal hyperalgesia (40). Higher ferritin levels have been linked to increased pain intensity (41). A recent single-nucleus characterization analysis revealed the effect of the Fth1 gene (encoding ferritin) on mechanical pain (42).

The two-sample method, which has a large amount of summary-level genetic data and may minimize potential confounding effects and reverse causation in observational research, is the study’s main contribution. In addition, to some extent, the GWAS research used in this MR study included European participants to eliminate the impact of population race. Furthermore, in a large GWAS of 48,972 European individuals, genetically predicted systemic iron status was identified, potentially reducing weak instrument bias.

There are several limitations to this study. First, all participants in our study were restricted to European ancestry to avoid any potential bias from ethnic differences. However, it is unknown whether this effect is suitable for other people. Second, our analyses were performed at the summary level, making stratified analyses by age, gender, and other relevant characteristics difficult. Third, we could not determine whether there were any non-linear relationships between the levels of systemic iron status biomarkers and the risk of back pain, such as a U-shaped relationship or a threshold effect. Fourth, all of these studies were small and underpowered to identify modest genetic influences on back pain risk (low power).

In conclusion, we presented genetic evidence for the potential correlations of iron status with increased risk of back pain in the European population. As a result, it is critical for European patients suffering from back pain to consider their systemic iron status. However, leave one out analysis revealed that when certain SNPs were excluded, the MR estimations were drastically reduced. To evaluate modest impacts, bigger MR studies or RCTs are required. Furthermore, researches including people of different ethnic origins based on individual-level data and research into the underlying process, were needed to reduce the risk of back pain.

## Data availability statement

The original contributions presented in this study are included in the article/Supplementary material, further inquiries can be directed to the corresponding author.

## Ethics statement

Written informed consent was obtained from the individual(s) for the publication of any potentially identifiable images or data included in this article. We used publicly available summary-level data. In the original studies, appropriate patient consent and ethical approval were obtained.

## Author contributions

YT, JW, MX, YS, and CC designed the study. YT, JW, MX, and HC collected and analyzed the data. YT, YS, HC, LW, and CC wrote the manuscript. All authors reviewed the manuscript and approved the final manuscript.

## Footnotes

1
http://www.nealelab.is/uk-biobank/
