# Supplementary material for: Causal associations of iron status and back pain risk: A Mendelian randomization study
Source: Front Nutr. 2022 Aug 10;9:923590. doi: 10.3389/fnut.2022.923590 (PMC9399786; doi:10.3389/fnut.2022.923590)
Supplement: Supplementary file 1 [file Data_Sheet_1.pdf]

frontiers

Supplementary Material

1 Supplementary Figures

1.1 Supplementary Figure 1

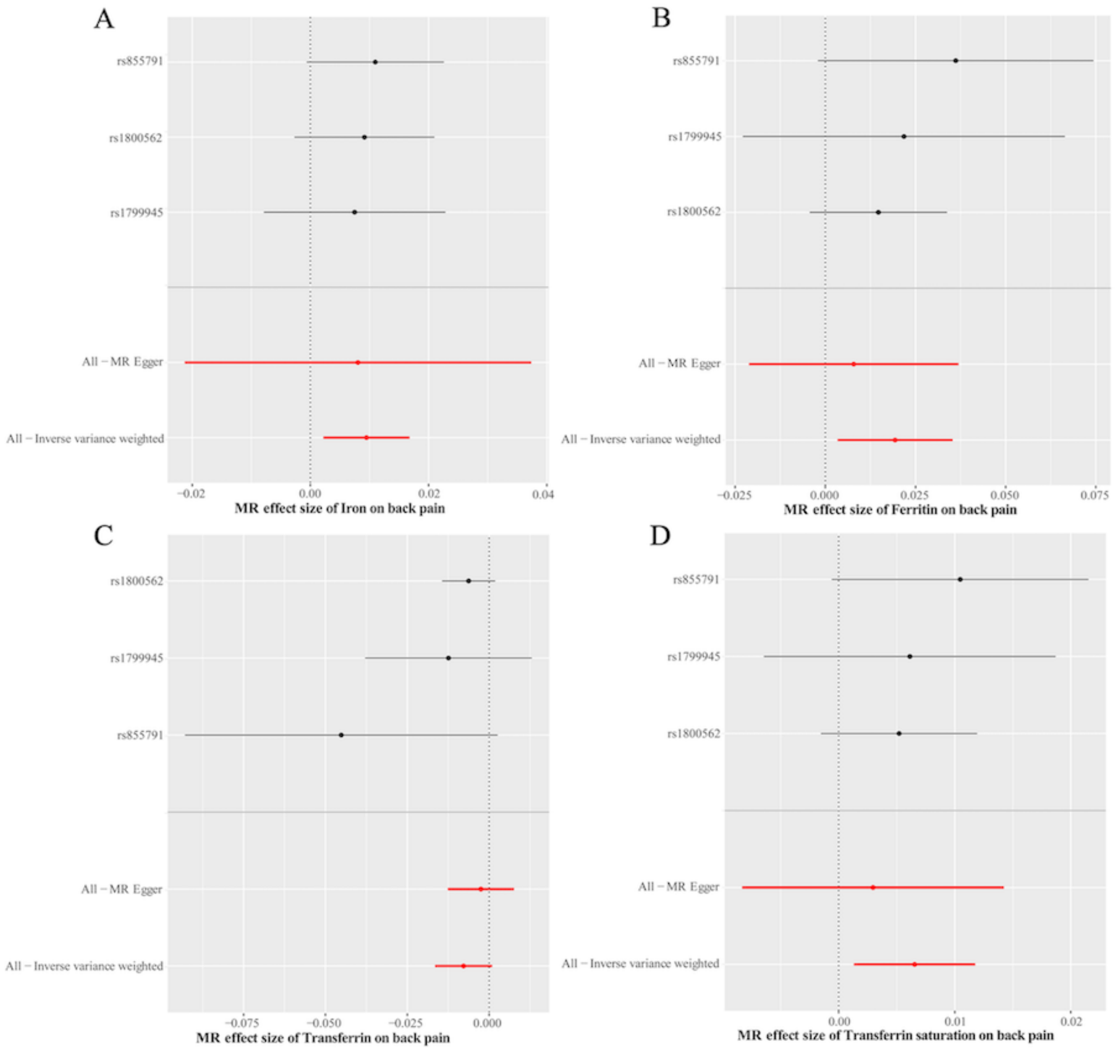

Supplementary Figure 1. Results of the single and multi-SNP analyses (conservative analysis) for the SNP effect of (A) Serum iron, (B) Ferritin, (C) Transferrin, and (D) Transferrin saturation on back pain.

1.2 Supplementary Figure 2

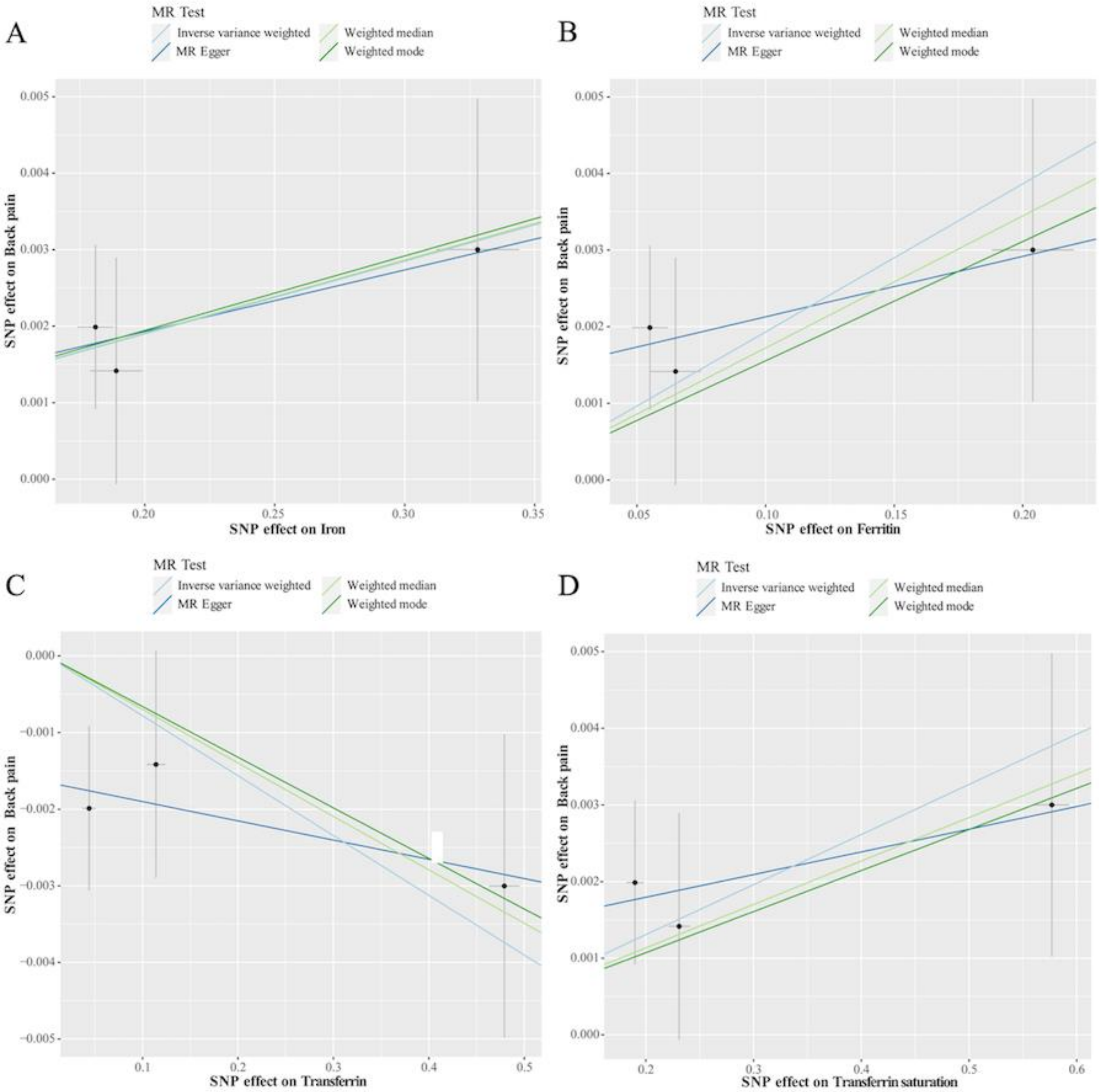

Supplementary Figure 2. A plot showing the effect sizes of the SNP-back pain (conservative analysis) associations (y-axis), (A) the SNP-Iron association (x-axis), (B) the SNP-Ferritin (x-axis), (C) Transferrin (x-axis), and (D) Transferrin saturation (x-axis) with standard error bars. The slopes of the lines correspond to causal estimates using IVW (light blue line), weighted median (light green line), MR-Egger (blue line), and weighted mode (green line) methods.

1.4 Supplementary Figure 3

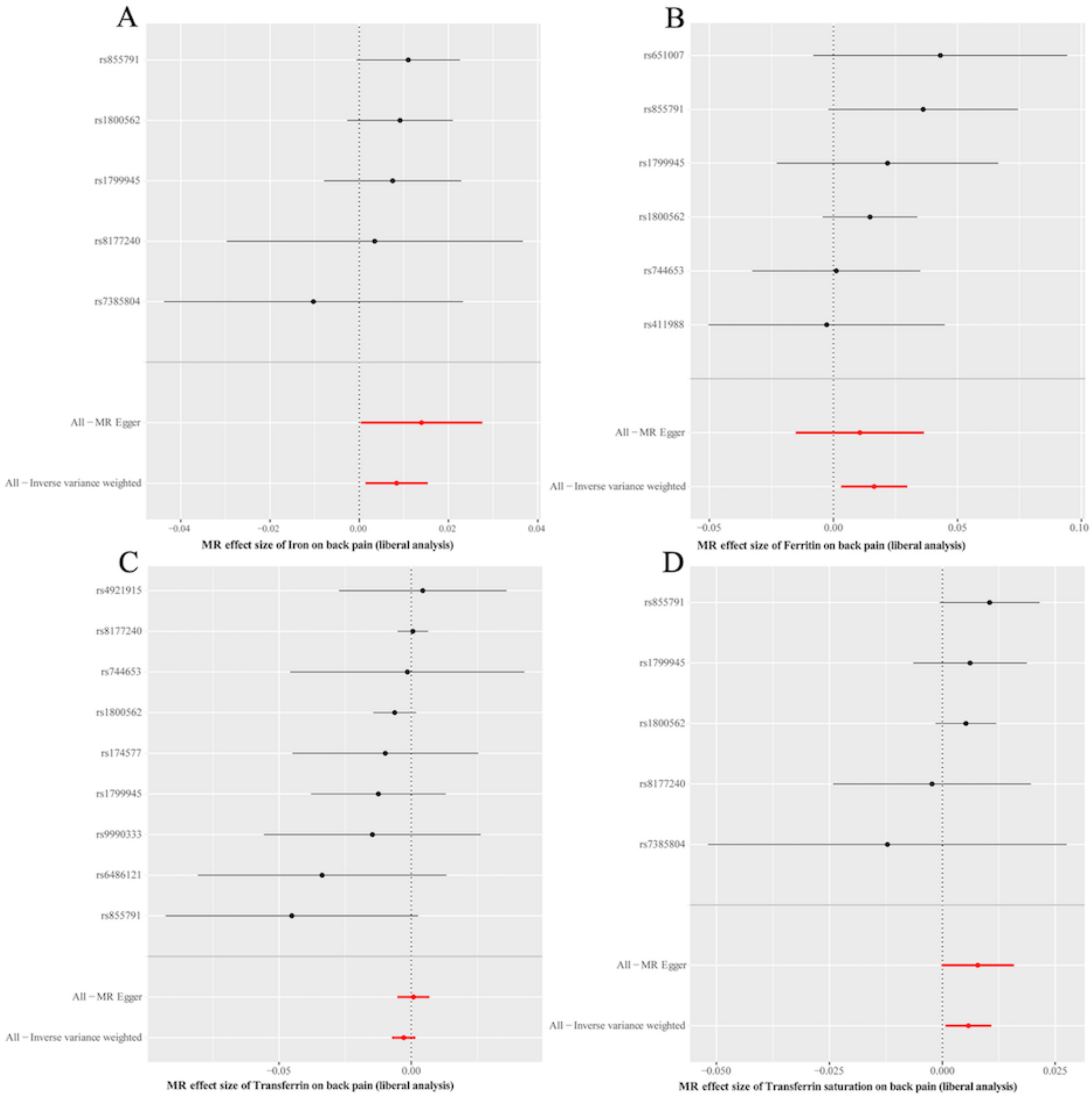

Supplementary Figure 3. Results of the single and multi-SNP analyses (liberal analysis) for the SNP effect of (A) Serum iron, (B) Ferritin, (C) Transferrin, and (D) Transferrin saturation on back pain.

1.5 Supplementary Figure 4

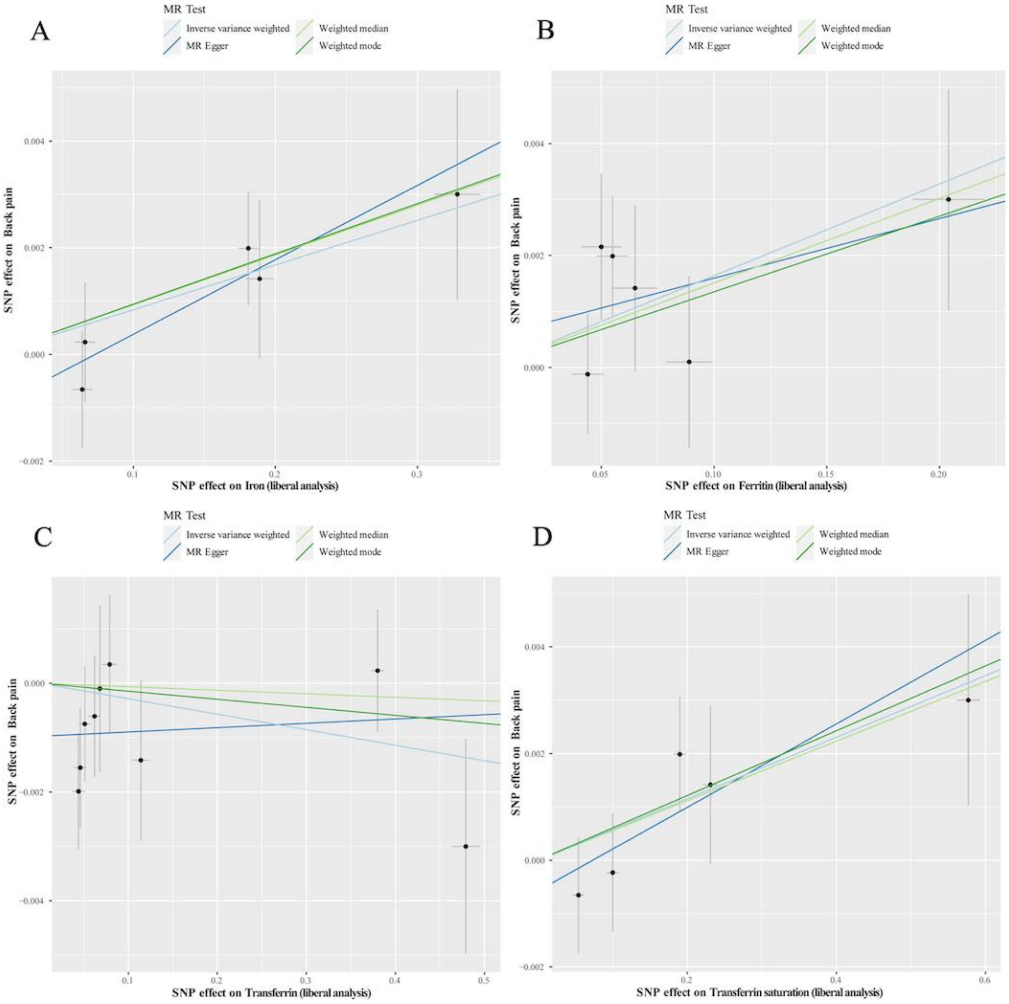

Supplementary Figure 4. A plot showing the effect sizes of the SNP-back pain (liberal analysis) associations (y-axis), (A) the SNP-Iron association (x-axis), (B) the SNP-Ferritin (x-axis), (C) Transferrin (x-axis), and (D) Transferrin saturation (x-axis) with standard error bars. The slopes of the lines correspond to causal estimates using IVW (light blue line), weighted median (light green line), MR-Egger (blue line), and weighted mode (green line) methods.

1.6 Supplementary Figure 5

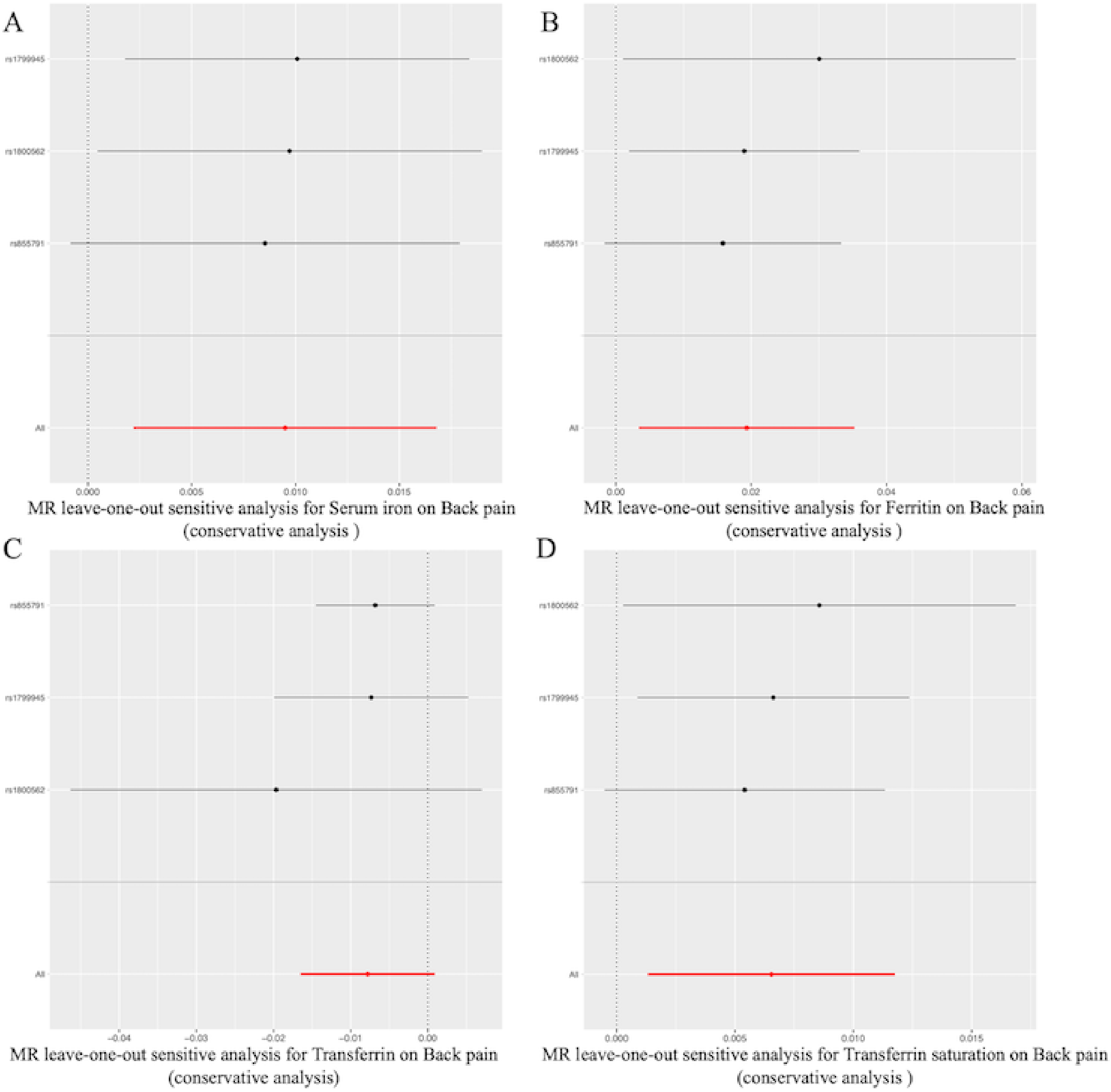

Supplementary Figure 5. Results of leave-one-out analysis (conservative analysis) for the SNP effect of (A) Serum iron, (B) Ferritin, (C) Transferrin, and (D) Transferrin saturation on back pain.

1.7 Supplementary Figure 6

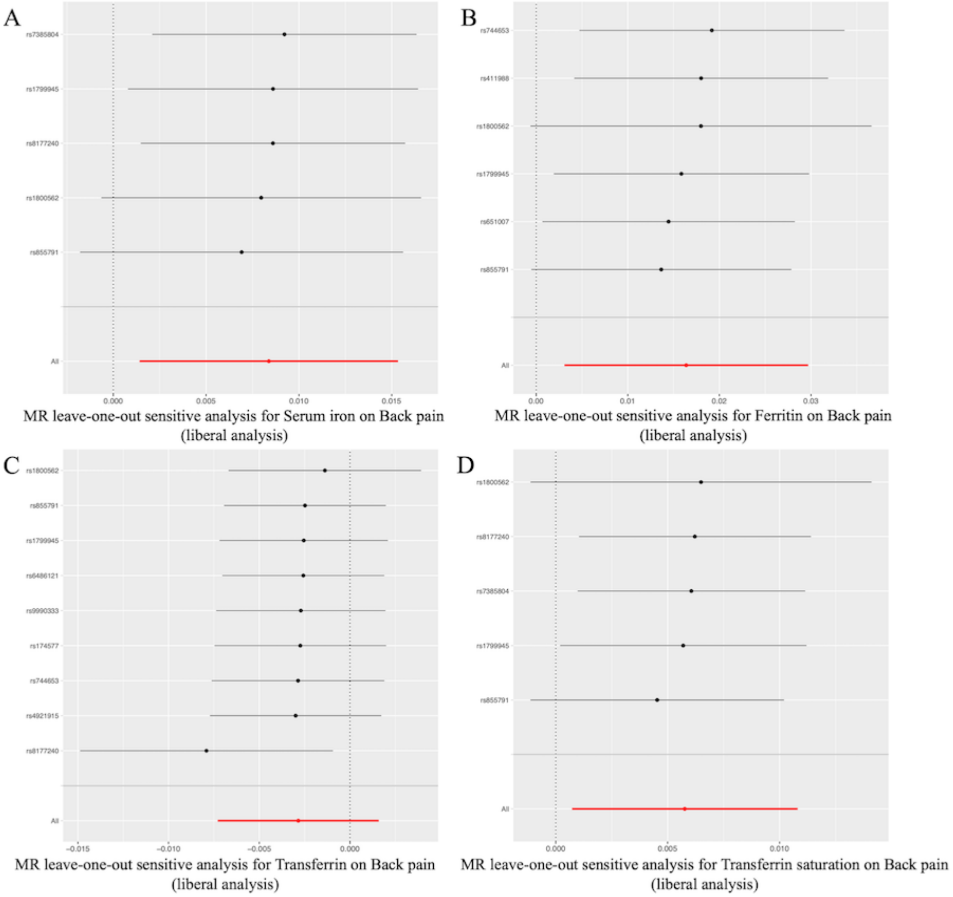

Supplementary Figure 6. Results of Leave-one-out analysis (liberal analysis) for the SNP effect of (A) Serum iron, (B) Ferritin, (C) Transferrin, and (D) Transferrin saturation on back pain.

1.9 Supplementary Figure 7

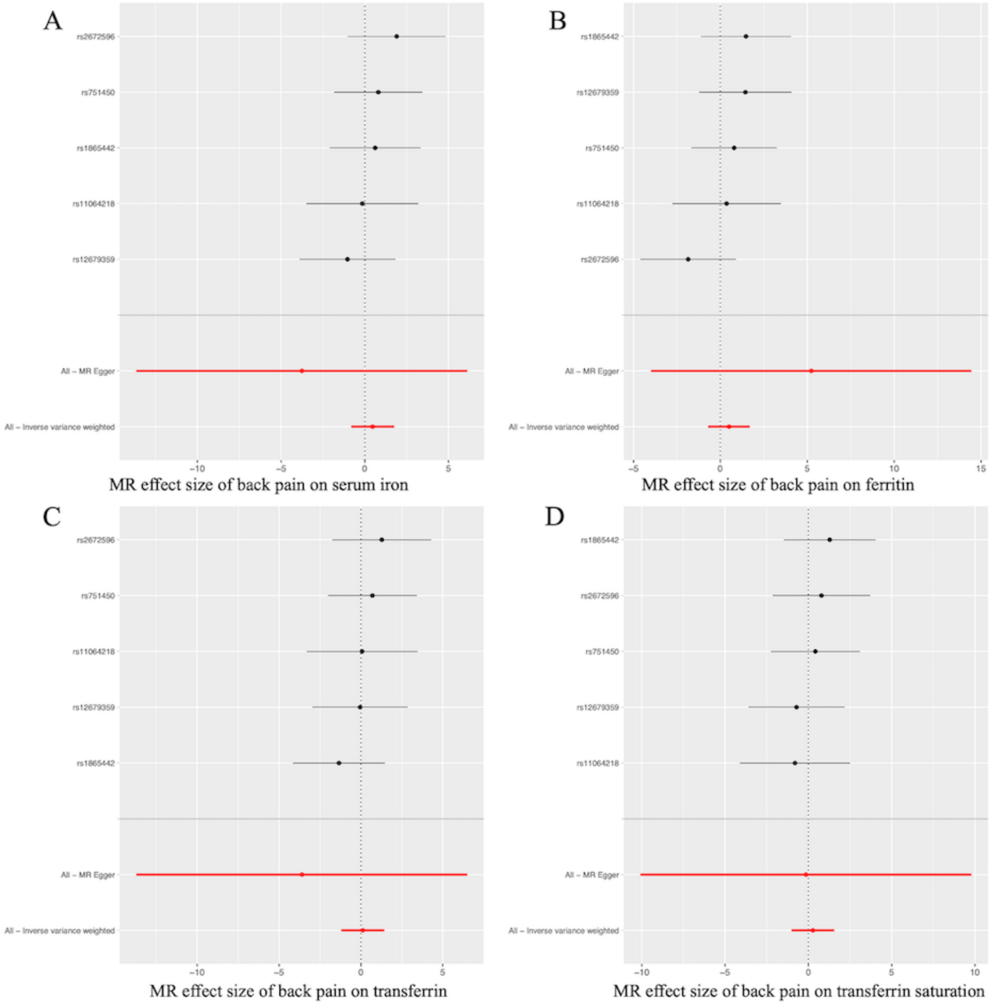

Results of the single and multi-SNP analyses for the SNP effect of back pain on (A) Serum iron, (B) Ferritin, (C) Transferrin, and (D) Transferrin saturation.

1.10 Supplementary Figure 8

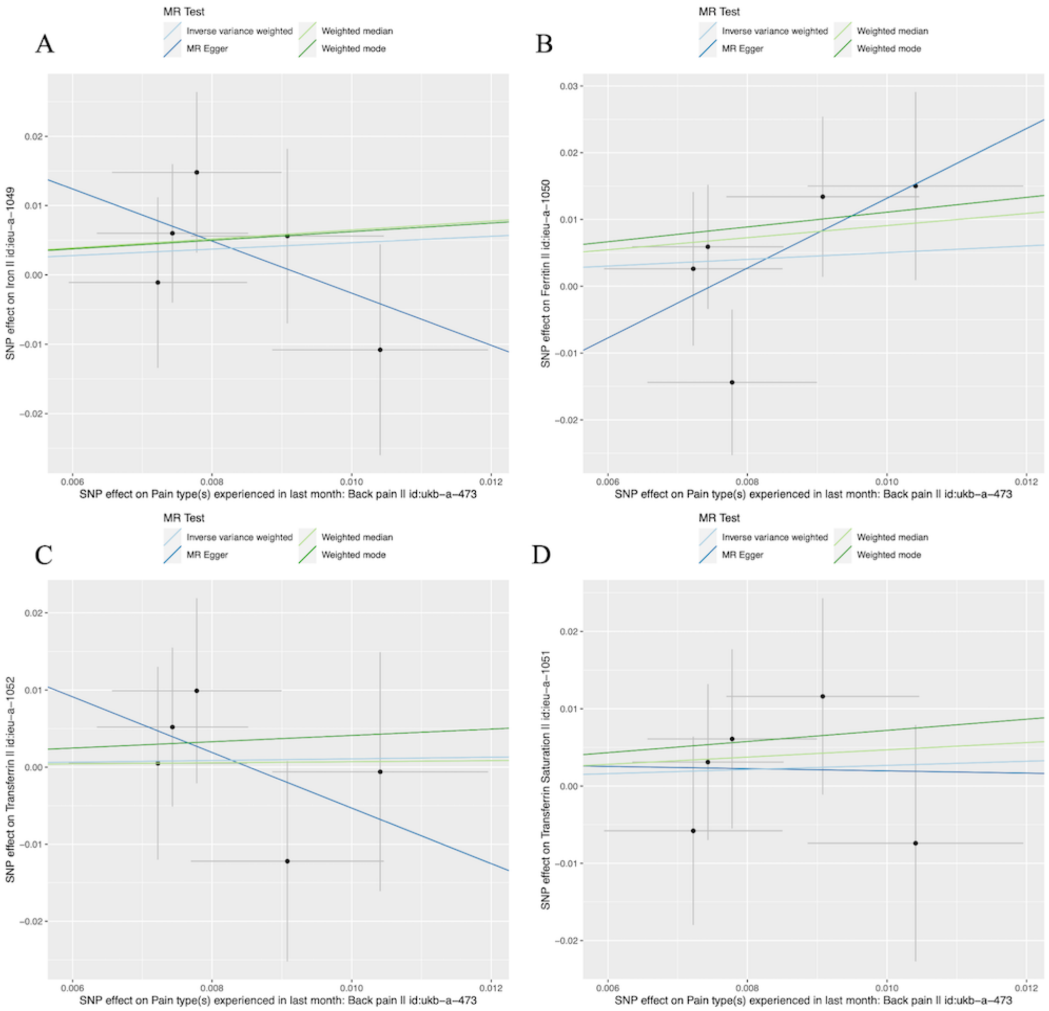

A plot showing the effect sizes of the back pain on systemic iron status associations, (A) Back Pain - Iron association, (B) Back Pain - Ferritin, (C) Back Pain - Transferrin, and (D) Back Pain -Transferrin saturation with standard error bars. The slopes of the lines correspond to causal estimates using IVW (light blue line), weighted median (light green line), MR-Egger (blue line), and weighted mode (green line) methods.

2 **Supplementary Table 1 The minimum and maximum true causal effects of iron status on back pain required to achieve 80% statistical power for inverse variance weighted (IVW) model.**

Analysis	Iron status	R^2	OR	Expected effects at 80% power
Conservative analyses	Iron	0.039	1.01	>1.057 or < 0.944
	Ferritin	0.007	1.02	>1.137 or < 0.871
	Transferrin	0.033	0.99	> 1.062 or < 0.939
	Transferrin saturation	0.073	1.01	> 1.042 or < 0.959
Liberal analyses	Iron	0.043	1.01	> 1.055 or < 0.947
	Ferritin	0.012	1.02	>1.105 or < 0.901
	Transferrin	0.104	1	>1.035 or < 0.965
	Transferrin saturation	0.079	1	>1.040 or < 0.960

3 **Supplementary Table 2 Cd for the instruments based on the best individual models with PP > 0.02**

**SNP**	**Nearest gene**	***Cd***			**Round (maxCD, digits = 3)**
		**1**	**2**	**3**	
rs1800562	HFE	0.047	0.013	0.257	0.257
rs1799945	HFE	0.011	0.009	0	0.011
rs855791	TMPRSS6	0.257	0.239	0.31	0.31
rs744653	SLC40A1	0.245	0	0	0.245
rs8177240	TF	0.007	0.007	0.051	0.051
rs9990333	TFRC	0	0.002	0.003	0.003
rs7385804	TFR2	0.009	0.092	0.021	0.092
rs4921915	NAT2	0	0	0.001	0.001
rs651007	ABO	0.128	0.001	0.001	0.128
rs6486121	ARNTL	0.004	0	0.003	0.004
rs174577	FADS2	0.001	0	0.001	0.001
**Threshold**		0.486	0.486	0.486	
